# Bioassay-guided isolation of two antiproliferative metabolites from *Pterocarpus indicus* Willd. against TGF-β-induced prostate stromal cells (WPMY-1) proliferation via PI3K/AKT signaling pathway

**DOI:** 10.3389/fphar.2024.1452887

**Published:** 2024-10-03

**Authors:** San Yoon Nwe, Tamonwan Uttarawichien, Teerawat Boonsom, Wisuwat Thongphichai, Peththa Wadu Dasuni Wasana, Boonchoo Sritularak, Witchuda Payuhakrit, Suchada Sukrong, Pasarapa Towiwat

**Affiliations:** ^1^ Department of Pharmacognosy and Pharmaceutical Botany, Faculty of Pharmaceutical Sciences, Chulalongkorn University, Bangkok, Thailand; ^2^ Center of Excellence in DNA Barcoding of Thai Medicinal Plants, Chulalongkorn University, Bangkok, Thailand; ^3^ Herb Guardian Co., Ltd., Nonthaburi, Thailand; ^4^ Animal Models of Chronic Inflammation-associated Diseases for Drug Discovery Research Unit, Chulalongkorn University, Bangkok, Thailand; ^5^ Department of Pharmacy, Faculty of Allied Health Sciences, University of Ruhuna, Galle, Sri Lanka; ^6^ Center of Excellence in Natural Products for Ageing and Chronic Diseases, Chulalongkorn University, Bangkok, Thailand; ^7^ Department of Pathobiology, Faculty of Science, Mahidol University, Bangkok, Thailand; ^8^ Chulalongkorn School of Integrated Innovation, Chulalongkorn University, Bangkok, Thailand; ^9^ Department of Pharmacology and Physiology, Faculty of Pharmaceutical Sciences, Chulalongkorn University, Bangkok, Thailand

**Keywords:** *Pterocarpus indicus*, angolensin, maackiain, benign prostatic hyperplasia, WPMY-1 cells, TGF-β

## Abstract

**Introduction:**

Benign prostatic hyperplasia (BPH) is the enlargement of the prostate gland, primarily occurring in aging men, in which transforming growth factor-beta (TGF-β) plays a critical role in prostate cell hyperproliferation and leads to uncomfortable urinary symptoms in BPH patients. *Pterocarpus indicus* Willd. is well known for its ethnopharmacological applications for treating ailments such as diuresis and bladder stones.

**Methods:**

This study aimed to examine the effect of *P. indicus* extract (PI extract) on TGF-β-induced WPMY-1 cell proliferation, followed by bioassay-guided fractionation to isolate the active metabolites. Angolensin (Ang) and maackiain (Mac) were isolated from bioassay-guided fractionation. Network analysis was performed to investigate the potential mechanisms. Furthermore, network analysis of the Ang-Mac combination in BPH highlighted the potential top ten pathways, including PI3K/AKT signaling pathway. Accordingly, subsequent investigation focused on evaluating the effect of PI extract, Ang, Mac, and Ang-Mac combination on the expression of PCNA, p53, and PI3K/AKT protein localization and expression.

**Results and discussion:**

Results revealed inhibition of cell proliferation in TGF-β-induced WPMY-1 cells, correlating with downregulated PCNA expression. While PI extract and Mac induced apoptosis via p53 upregulation, Ang and Ang-Mac combination did not significantly affect apoptosis through the p53 pathway. Additionally, both metabolites exhibited potent inhibition of p-PI3K and p-AKT protein localization and expression in the nucleus of TGF-β-induced WPMY-1 cells. This study suggests that PI extract, Ang, and Mac are promising compounds for treating BPH, as evidenced by *in silico* and *in vitro* studies. Additionally, Ang and Mac could be used to standardize PI extract in future investigations.

## 1 Introduction

The non-malignant enlargement of the prostate, also known as benign prostatic hyperplasia (BPH), which its prevalence increases with age, approximately 8% in the fourth decade of life and 50% in 50–60 years old men, and increases up to 80% in the ninth decade (Berry et al., 1984; Kok et al., 2009; Awedew et al., 2022). The majority of clinical BPH cases were developed in the transition zone of the prostate (Sellers et al., 2024), which causes the constriction of the urethra as it passes through the prostate, causing bladder outlet obstruction. This condition leads to various urinary complications known as lower urinary tract symptoms (LUTS) including nocturia, intermittent, urgency, frequency, and incomplete emptying (Roehrborn, 2005; Agarwal et al., 2014).

Pathological BPH is characterized by hyperplastic epithelial and stromal cells forming nodules in the prostate. The earliest nodules appearing in BPH are typically found in the periurethral area and consist mainly of stromal cell (Nicholson and Ricke, 2011). Overall, stromal and epithelial components make up about 80% and 20% of the hyperplastic volume of the prostate gland, respectively (Bartsch et al., 1979; Lepor, 2007). Particularly, stromal cells exhibit higher levels of proliferation than epithelial cells, indicating that the BPH is characterized by stromal-dominant proliferation (Hata et al., 2023). Notably, transforming growth factor-beta (TGF-β) plays a pivotal role in maintaining the equilibrium of cell proliferation and apoptosis in the prostate (Baba et al., 2022). Once the tissue is stimulated by aging, hormonal alterations, inflammation, or metabolic syndromes, the tissue loses this balance, resulting in an increase in TGF-β levels, leading to prostate cell hyperproliferation (Li et al., 2020; Wang et al., 2017a). Therefore, the modulation of TGF-β activation and understanding the molecular pathways involved in proliferation and programmed cell death in the prostate is imperative for the development of new therapies (Minutoli et al., 2016).

Alpha-blockers used alone or in conjunction with 5α-reductase inhibitors, are proved to be effective in managing BPH (Vuichoud and Loughlin, 2015). Nevertheless, they often lead to adverse effects such as impaired ejaculation, sexual dysfunction, nasal stuffiness, asthenia, postural hypotension, loss of appetite, and dizziness (Lepor, 2007; McConnell et al., 2003). Hence, the interest in phytotherapy has been growing to investigate new therapeutic alternatives for BPH. *Pterocarpus indicus* Willd. (PI), commonly called rosewood, is widely distributed in Southeast Asia and is well known for its ethnopharmacological applications. The decoction and crude extracts of *P. indicus* have been used in the management of common ailments such as diuresis, bladder stones, ulcers, diarrhea, and dysentery (Ragasa et al., 2005). Notably, the phytoconstituents responsible for relieving BPH have not been investigated yet. WPMY-1 is a human prostate myofibroblast cell line commonly employed for *in vitro* evaluation of the anti-BPH activity. Therefore, this study aimed to explore the antiproliferative activity of *P. indicus* crude extract on TGF-β-induced WPMY-1 cell proliferation, conduct bioassay-guided fractionation to isolate active metabolites responsible for the antiproliferative activity and investigate the potential mechanisms of the PI extract and isolated secondary metabolites using *in silico* and *in vitro* studies.

## 2 Materials and methods

### 2.1 Chemicals and reagents

Silica gel (70–230 and 230–400 mesh size), thin layer chromatography (TLC) silica gel F245 plate, and NMR solvents (chloroform-*d*_*1*_, acetone-*d*_*6*_) were purchased from Merck KGaA (Darmstadt, Germany). Sephadex LH-20 was obtained from Pharmacia Biotech AB (Uppsala, Sweden). Ethanol (EtOH), methanol (MeOH), acetone, ethyl acetate (EtOAc), and dichloromethane (CH_2_Cl_2_) were purchased from a local chemical company and distilled before use. 3-(4,5-dimethylthiazol-2-yl)-2,5-diphenyl tetra-sodium bromide (MTT) reagents (cat. no. M6494), 1% penicillin–streptomycin (cat. no. 1IVG7-15,140–122), 1% GlutaMAX (cat. no. 1IVG7-35,050–061), 10% fetal bovine serum (FBS) (cat. no. F0804), and Dulbecco’s modified Eagle’s medium (DMEM) were purchased from Thermo Fisher Scientific (Massachusetts, United States). Recombinant human transforming growth factor 1 (TGF-β1) (cat. no. 781804) and propidium iodide (PI) and Annexin V-fluorescein isothiocyanate (FITC) staining kit (cat. no. 640906), and PCNA antibody (cat. no. 307901) were received from Biolegend, United States. p53 (cat. no. ab131442) and phosphorylated-AKT antibodies (cat. no. ab38449) were received from Abcam, United Kingdom. Phosphorylated-PI3K antibody (cat. no. CSB-PA030058) was purchased from Cusabio, United States. Total-PI3K antibody (cat. no. 05-212) was purchased from Merck KGaA, Germany. Total-AKT antibody (cat. no. 9272S) was obtained from Cell Signaling Technology, United States. β-actin antibody (cat. no. bs-0061R) was purchased from Bioss, United States. *Pterocarpus indicus* was collected from Bangkok, Thailand. The identification of plants was done by Professor Boonchoo Sritularak, Ph. D, through comparison with the Botanical Garden Organization’s database. A voucher specimen (SS-1105) has been deposited at the Herbarium of the Faculty of Pharmaceutical Sciences, Chulalongkorn University, Bangkok, Thailand.

### 2.2 Cell culture

The normal human prostate stromal cell line WPMY-1 was obtained from the American Type Culture Collection (ATCC^®^ CRL2854™). The cells were cultured in Dulbecco’s Modified Eagle’s Medium supplemented with 10% fetal bovine serum, 1% GlutaMax, and 1% penicillin-streptomycin. The cells were maintained at 37°C in a humidified atmosphere with a 5% CO_2_ incubator (Thermo Scientific™ Steri-Cycle i160).

### 2.3 Cell viability and cell proliferation assay

Cell viability of WPMY-1 cells was assessed using the MTT assay to determine the maximum non-toxic concentration of the tested secondary metabolites (Riss et al., 2016). The cells were seeded in 96-well plates at 1 × 10^4^ cells/well density and incubated at 37°C with 5% CO_2_ for 24 h in a humidified incubator. After incubation, the seeded cells were treated with the extract or metabolites for 24 h. After treatment, serum-free media containing MTT solution were added and incubated for 4 h, followed by DMSO to dissolve the formazan crystals. The absorbance was measured at 570 nm using a microplate reader (CALIOSTAR). WPMY-1 cells were treated with or without TGF-β, along with extract or metabolites, for 24 h to assess the antiproliferative effect of extract or metabolites. Then, cell proliferation was evaluated using the MTT assay as previously described.

### 2.4 Bioassay-guided isolation of bioactive metabolites from the *Pterocarpus indicus* extract

The dried heartwood of *P. indicus* was pulverized to fine powder and repetitively macerated with EtOAc at room temperature until exhausted. The extract was then concentrated under reduced pressure using a rotatory evaporator at 40°C to yield the EtOAc crude extract (130 g). The resulting crude extract was screened for its antiproliferative activity on the TGF-β-induced WPMY-1 cell line. The extract was then fractionated on a silica gel column (10 cm × 22 cm) eluted with a gradient mixture of n-hexane: EtOAc (1:0 to 0:1) followed by EtOAc:MeOH (20:1 to 1:1). Three fractions (A–C) were collected and investigated for the inhibition effect on prostate cell proliferation. The fractions showing the antiproliferative activity were used for further purification using silica column chromatography and Sephadex LH20 column, eluted with various organic solvents. The structures of isolated compounds were elucidated and characterized by spectroscopic techniques, including mass spectrometry (MS) and nuclear magnetic resonance (NMR) spectroscopy.

### 2.5 Network analysis

The canonical SMILES of Angolensin (Ang) and Maackiain (Mac) were obtained from the PubChem database (https://pubchem.ncbi.nlm.nih.gov). Potential targets of the Ang and Mac were obtained from web-based prediction tools including Swiss target Prediction (http://www.swisstargetprediction.ch/), SEA SearchServer (https://sea.bkslab.org/), PharmMapper (https://www.lilab-ecust.cn/pharmmapper/) (Wang et al., 2017b; Keiser et al., 2007). Furthermore, genes related to the BPH were obtained from three databases, including the DisGeNET database (https://www.disgenet.org/), OMIM database (https://www.omim.org/), and GeneCards (Score ≥ 10) (https://www.genecards.org/). The data retrieval process was conducted and completed on 12 June 2023.

The gene symbols of potential target genes were validated using the UniProt database (https://www.uniprot.org/). After the validation process, the likely target genes of the metabolites and BPH were combined, and any duplicate entries were then removed separately for both sets of targets. The intersection between the potential target genes of the Ang-Mac combination and BPH was identified by Venny 2.1 (https://bioinfogp.cnb.csic.es/tools/venny/). Then, the common genes were used as potential targets of the Ang-Mac combination in BPH to construct the protein-protein interaction (PPI) network using the STRING database (https://string-db.org/). The resulting PPI network between the Ang-Mac combination and BPH was sent to the Cytoscape (3.10.1) for further analysis of PPI. The top 10 genes with the highest level of interaction were selected by the cytoHubba plugin. Kyoto Encyclopedia of Genes and Genomes (KEGG) and Gene Ontology (GO) pathway analyses were performed to elucidate the potential mechanism of action (Kanehisa and Goto, 2000). The online bioinformatics data analysis tool (https://www.bioinformatics.com.cn/) was used to conduct and visualize the GO and KEGG enrichment analysis.

### 2.6 Flow cytometry analysis

The apoptotic cell population was identified using flow cytometry with a FITC Annexin V staining kit. WPMY-1 cells (3.0 × 10^4^) were seeded into 24-well plates and incubated for 24 h at 37°C with 5% CO_2_. Subsequently, the media was replaced with serum-free media containing TGF-β (5 ng/mL), with or without PI extract 50 μg/mL, Ang 154 ng/mL, Mac 66 ng/mL, or Ang-Mac combination (154 + 66 ng/mL). After 24 h incubation, the cells were trypsinized, washed with phosphate-buffered saline (PBS), and centrifuged at 2,000 rpm for 5 min. The cell pellet was resuspended in a binding buffer with annexin V conjugated with FITC, PI conjugated with phycoerythrin and incubated in the dark for 15 min. Labeled cells were evaluated using FACScan (Becton, Dickinson and Company, Franklin Lakes, NJ, United States).

### 2.7 Indirect immunofluorescence assay

Protein localization was assessed using indirect immunofluorescence. WPMY-1 cells were seeded in 96-well plates at a density of 3 × 10^3^ cells/well. After 24 h incubation period at 37°C with 5% CO_2_, serum-free media containing 5 ng/mL TGF-β, with or without the PI extract 50 μg/mL, Ang 154 ng/mL, Mac 66 ng/mL, or Ang-Mac combination (154 + 66 ng/mL) were added. Following an additional 24 h incubation, the cells were permeabilized with 0.25% Triton X-100 and fixed with cold methanol. Subsequently, primary antibodies, anti-PCNA (1:1,000), anti-phosphorylated-PI3K (p-PI3K) (1:1,000), and anti-phosphorylated-AKT (p-AKT) (1:1,000) were added and incubated for 1.5 h. This was followed by adding a FITC-conjugated secondary antibody with an incubation period of 30 min. Nuclear counterstaining with DAPI (1:500 dilution) was performed for 15 min. The cells were examined using a fluorescence microscope (Nikon Eclipse Ni model with Nikon Digital Sight 10 camera).

### 2.8 Western blot analysis

WPMY-1 cells were seeded into 6-well plates at 3 × 10^5^ cells/well density and incubated for 24 h. Subsequently, the cells were treated with serum-free media containing 5 ng/mL TGF-β, either with or without PI extract 50 μg/mL, Ang154 ng/mL, Mac 66 ng/mL, or Ang-Mac combination (154 + 66 ng/mL). After each treatment, the cells were washed twice with PBS and lysed with radioimmunoprecipitation assay (RIPA) buffer supplemented with 1% protease inhibitor. Protein concentration was determined using the Bradford protein assay. The cell lysates were mixed with 6X SDS-PAGE loading buffer, and equal amounts of protein were heated at 95°C for 5 min before being separated on 12% SDS-polyacrylamide gels. The proteins were transferred onto nitrocellulose membranes, blocked in Tris-buffered saline with 0.05% Tween-20 (TBS-T) containing 5% non-fat dried skim milk for 1 h. After washing with TBS-T, the membranes were incubated overnight at 4°C with primary antibodies, including total-PI3K (t-PI3K) (1:1,000), p-PI3K (1:1,000), total-Akt (t-Akt) (1:1,000), p-Akt (1:1,000), p53 (1:1,000), PCNA (1:1,000), and β-actin (1:2,000). Following further washing with TBS-T, the membranes were incubated with secondary antibodies (goat anti-mouse or rabbit conjugated-horseradish peroxidase enzyme, 1:1,000) for 1 h. After additional washes with TBS-T, the signals were visualized using the Molecular Imager^®^ ChemiDoc™ XRS + Imaging System (Bio-Rad). The intensities of the protein bands were quantified using ImageJ software.

### 2.9 Statistical analysis

The mean ± standard deviation (SD) of each result was presented. GraphPad Prism software was utilized to analyze the biological assay using one-way analysis of variance (ANOVA) and Tukey’s *post hoc* test. Statistical significance was considered when *p* < 0.05.

## 3 Results

### 3.1 Bioassay-guided isolation of bioactive metabolites from *Pterocarpus indicus* extract

**FIGURE 1:**
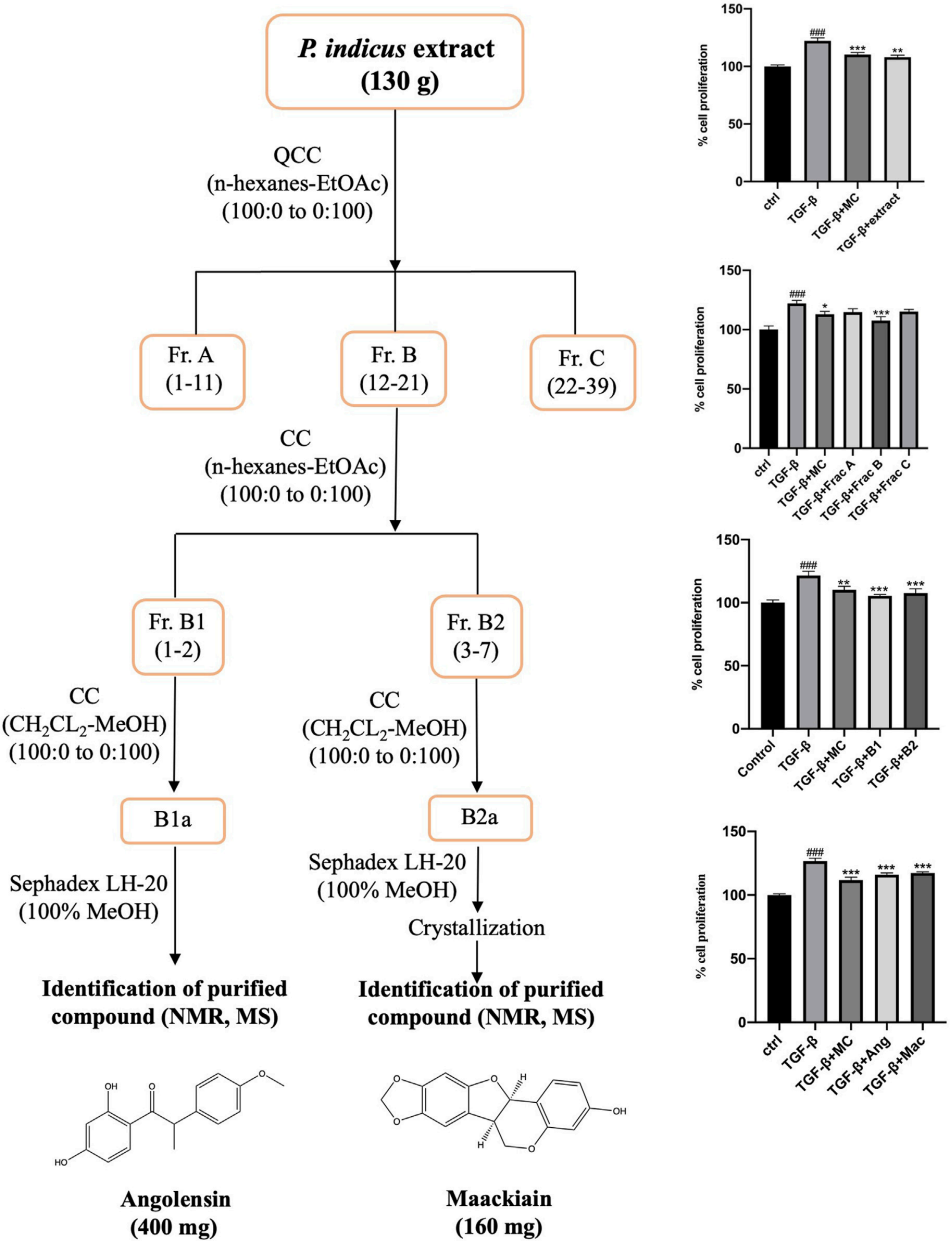
Flow chart for bioassay-guided isolation of metabolites from *Pterocarpus indicus* extract based on the antiproliferative screening of extract, fractions, and isolated metabolites. The percentage of cell proliferation is presented as mean ± SD, ^###^ *p* < 0.001 compared to control, ***p < 0.001, **p < 0.01, and *p < 0.05 compared to TGF-β.

Based on these cytotoxicity studies, we have selected maximum non-toxic concentrations of PI extract, fractions, and metabolites for bioassay-guided isolation assay (Supplementary Figure S1). To isolate the antiproliferative metabolites from the heartwood of PI extract, bioassay-guided isolation employing silica gel column chromatography, Sephadex LH20, and crystallization, and MTT assay were used as described in Materials and Methods (Figure 1). The PI extract (50 μg/mL) was screened for its antiproliferative activity on the TGF-β induced WPMY-1 cells. The cell proliferation was significantly increased with the treatment of TGF-β (5 ng/mL) compared to 100% of the control. However, the proliferation of cells was significantly decreased after treatment with PI extract (50 μg/mL) when compared to the TGF-β treatment group. This bioactive extract (130 g) was then fractionated on a silica gel column (10 cm × 22 cm) eluted with a gradient mixture of n-hexane:EtOAc (1:0 to 0:1) followed by EtOAc:MeOH (20:1 to 1:1). Three fractions (A–C) were collected and investigated for the inhibition effect on prostate stromal cell proliferation. Fraction B (1 μg/mL), which showed significant antiproliferative activity was then proceeded for further separation by column chromatography. Fraction B (16.4 g) was then separated on a silica gel column eluted with n-hexane: acetone (9:1) to give two subfractions (B1-B2). Both B1 (5 μg/mL) and B2 (1 μg/mL) subfractions also significantly inhibited the proliferation of WPMY-1 cells. As further separation was needed to isolate the active metabolites, subfraction B1 (9.1 g) was fractionated again by column chromatography (silica gel, CH_2_CL_2_:MeOH, gradient). Then, it was purified on a Sephadex LH20 column eluted with MeOH to give PI-B1 (400 mg). Subfraction B2 was further separated on CC using CH_2_CL_2_-MeOH as the eluent. Afterward, purification was conducted with Sephadex LH20, eluted with MeOH, and then recrystallized from MeOH to give PI-B2 (160 mg). The structures of isolated compounds were elucidated and characterized by spectroscopic techniques, including MS and NMR (Supplementary Figures S2–S5). By the comparison with previous literature, PI-B1, and PI-B2 were identified as angolensin (Salakka and Wähälä, 1999) and maackiain (Peng et al., 2016), respectively (Supplementary Tables S1, S2).

### 3.2 Network analysis between angolensin-maackiain combination and benign prostatic hyperplasia

**FIGURE 2:**
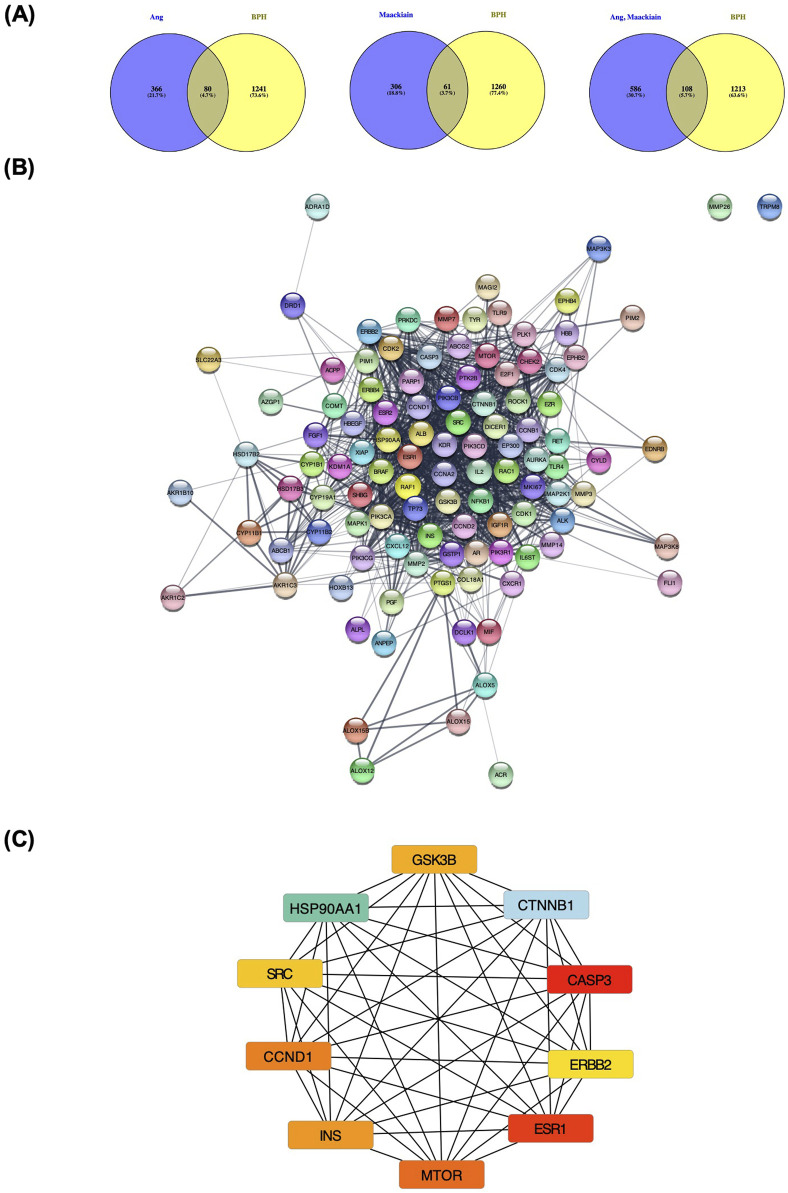
Network pharmacology analysis of angolensin-maackiain combination on BPH. **(A)** The intersection between the isolated metabolites (Ang, Mac, and Ang-Mac combination) and BPH. **(B)** The protein-protein intersection network of 108 common genes between Ang-Mac combination. **(C)** The protein-protein interaction between the top 10 proteins, including Catenin Beta 1 (CTNNB1), Caspase 3 (CASP3), Estrogen Receptor 1 (ESR1), Heat shock protein HSP 90-alpha (HSP90AA1), Mammalian target of rapamycin kinase (MTOR), Cyclin D1 (CCND1), Insulin (INS), Glycogen synthase kinase-3 beta (GSK3B), Proto-oncogene tyrosine-protein kinase Src (SRC), and Receptor tyrosine-protein kinase erbB-2 (ERBB2).

Network analysis was performed to predict the potential mechanism of angolensin and maackiain in BPH (Figure 2). The Venn diagram shows that the intersection target genes between BPH and Ang, Mac, and the Ang-Mac combination were 80, 61, and 108 respectively. The 108 common genes of BPH and Ang-Mac combination were imported into the STRING database to obtain PPI (Figure 2A). The PPI contained 108 nodes and 1,146 edges. Calculated values for the average node degree and average node clustering coefficients were 21.2 and 0.648, respectively, indicating the average number of protein interactions in the network and the health of the nodes that are connected to the network. The PPI network was further analyzed and visualized using Cytoscape 3.9.2 (Figure 2B). The cytoHubba plugin was used to investigate the key targets. The top ten genes were CTNNB1, CASP3, ESR1, HSP90AA1, MTOR, CCND1, INS, GSK3B, SRC, and ERBB2, respectively (Figure 2C).

**FIGURE 3:**
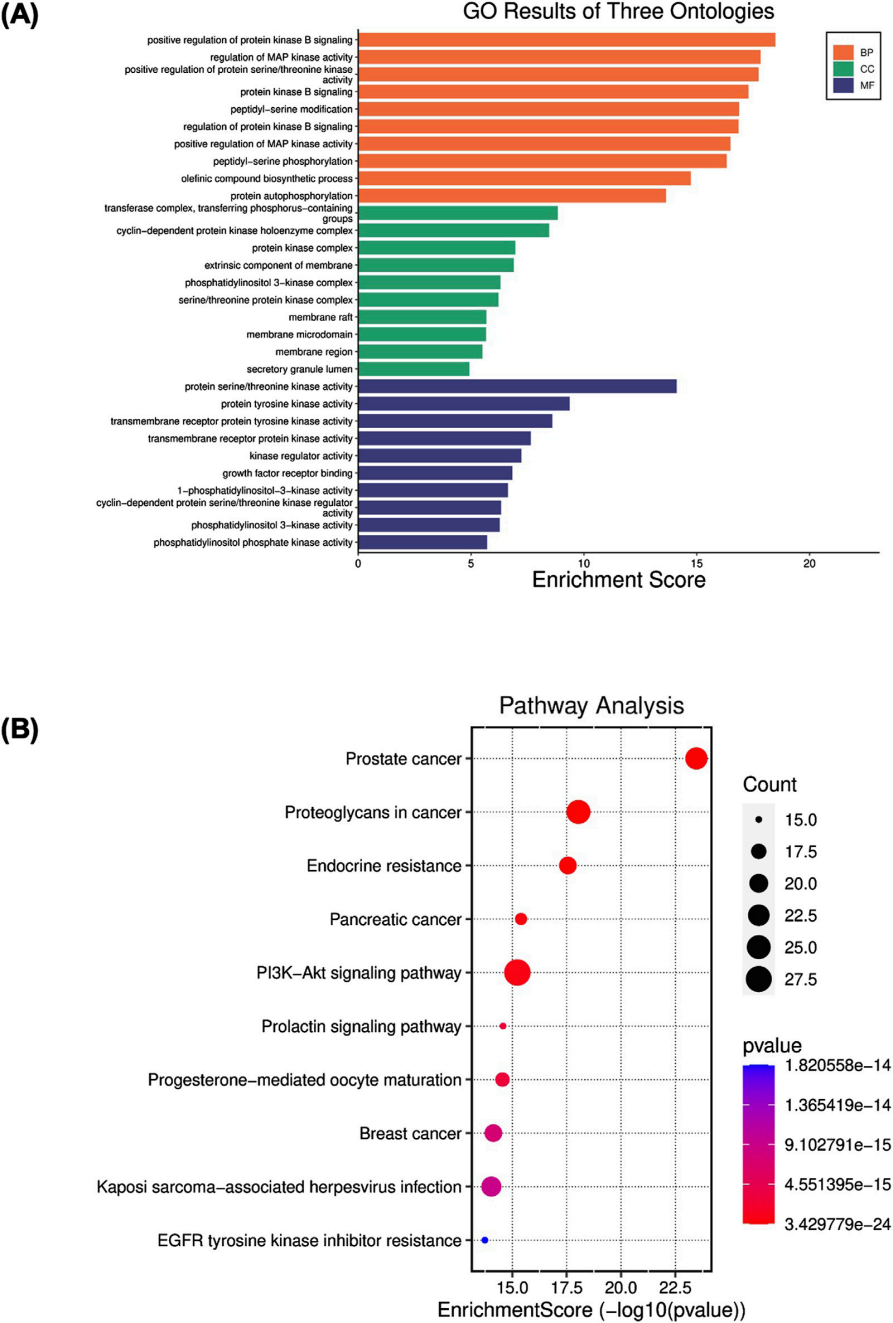
**(A)** Gene Ontology (GO) enrichment analysis of angolensin-maackiain combination on BPH. **(B)** Kyoto Encyclopedia of Genes and Genomes (KEGG) pathway enrichment analyses.

According to the results of GO analysis, regulation of protein kinase B signaling, regulation of MAP kinase activity, positive regulation of protein serine/threonine kinase, peptidyl serine modification, and phosphorylation, protein autophosphorylation were the critical processes involved in the Biological process (BP) enrichment. In the Molecular functions (MF) enrichment, it is primarily involved protein kinase activity, growth factor receptor binding activity, and phosphatidylinositol 3-kinase activity at the molecular level. Cell complex is associated mainly with the transferase complex, protein kinase complex, extrinsic component of membrane, membrane microdomain, and secretory granule lumen. They may have significant relevance to the pathological progression of BPH (Figure 3A).

KEGG analysis was conducted to illustrate the possible anti-BPH mechanism of Ang-Mac combination. The results revealed that the most involved target genes are closely related to cancer pathways, including prostate cancer, proteoglycan in cancer, endocrine resistance, pancreatic cancer, PI3K-AKT signaling pathway, breast cancer, and EGFR tyrosine kinase inhibitor resistance (Figure 3B). These signaling pathways are mostly linked to apoptosis and cell proliferation processes. Among these pathways, PI3K-AKT involves in the cell proliferation and apoptosis, which might be linked to the development of BPH (Sreenivasulu et al., 2018). In our study, we explored the effect of the PI extract, Ang, Mac, and Ang-Mac combination on the expression of proteins involved in the PI3K-AKT signaling pathway in the subsequent *in vitro* analysis. In the pathway enrichment analysis, the false discovery rate (FDR)-adjusted *p*-value of all the top ten terms were lower than 0.05 (Supplementary Tables S3, S4), suggesting that lower probability of false positives for less than 5% of the significant terms.

### 3.3 Antiproliferative activity of PI extract, isolated compounds on TGF-β induced WPMY-1 cell proliferation

**FIGURE 4:**
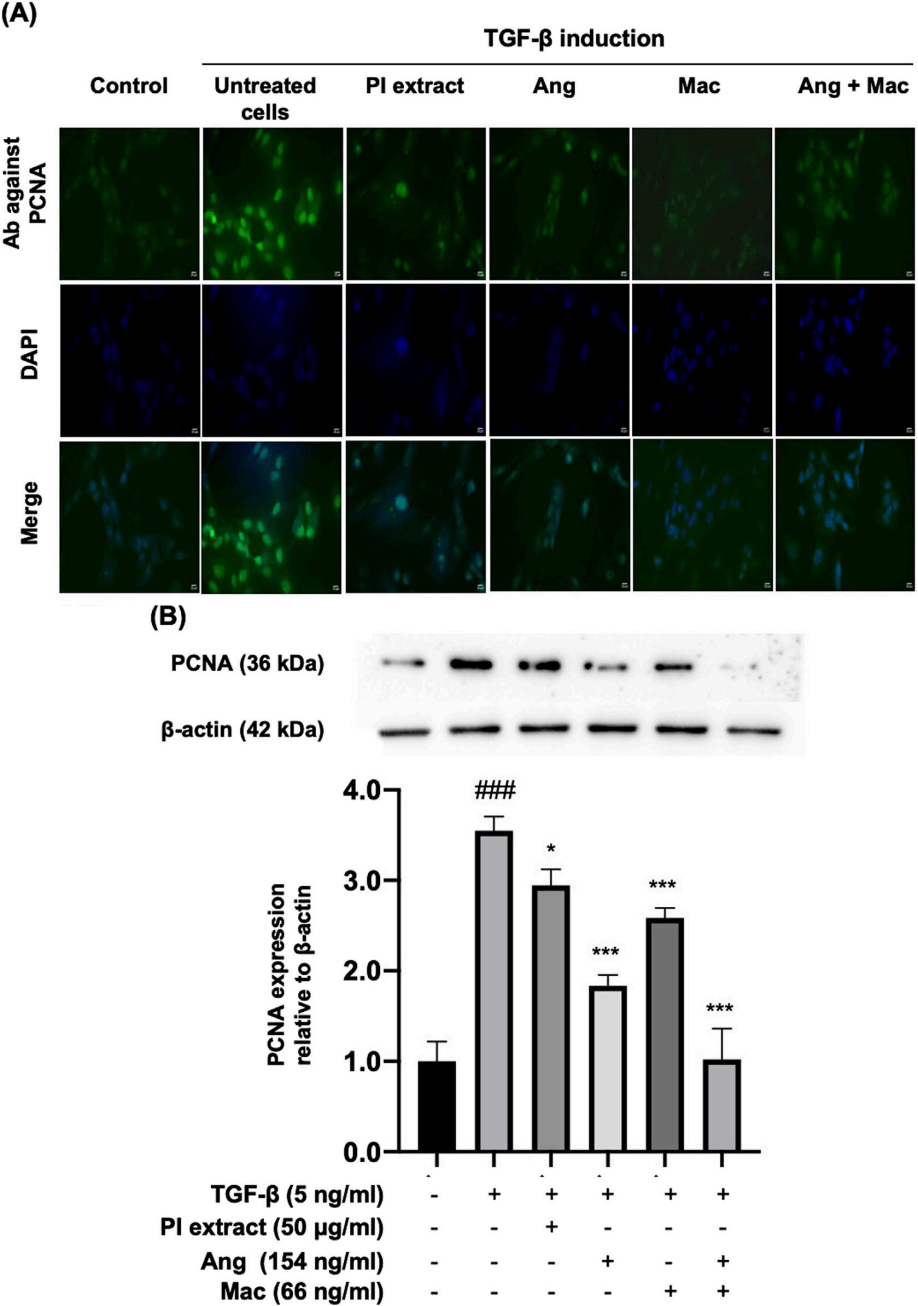
The effect of PI extract, Ang, Mac, and Ang-Mac combination on TGF-β-induced PCNA expression. WPMY-1 cells were treated with 5 ng/mL of TGF-β alone or in combination with PI extract (50 μg/mL), Ang (154 ng/mL), Mac (66 ng/mL), and Ang-Mac combination (154 + 66 ng/mL). **(A)** Immunofluorescence images reveal the population of WPMY-1 cells, with green signals indicating PCNA protein expression in the nucleus, while blue represents nuclear staining with DAPI. Scale bar: 50 μm, magnification: ×400. **(B)** Protein bands of PCNA and expression of PCNA are normalized to β-actin and shown as relative density compared to the control. Data are presented as mean ± SD, ^###^ *p* < 0.001 compared to control, ***p < 0.001 and *p < 0.05 compared to TGF-β.

The study tested whether PI extract, Ang, Mac, and Ang-Mac combination treatment inhibits the PCNA protein expression (Figure 4). As a result of the immunofluorescent staining assay revealed, the expression of PCNA in the nucleus of the cells as green signals significantly increased in the presence of TGF-β. Notably, after treatment with PI extract, Ang, Mac, and Ang-Mac combination, PCNA expression in TGF-β-induced WPMY-1 cells was significantly decreased (Figure 4A). In addition, the Western blot analysis was employed to confirm the expression of PCNA protein. The cells treated with the PI extract (50 μg/mL) exhibited a significant decrease in the PCNA expression, as well as decrease in response to the treatment with Ang (154 ng/mL), Mac (66 ng/mL), and Ang-Mac combination compared to that of the TGF-β-induced WPMY-1 cells (Figure 4B). Therefore, the PI extract, Ang, and Mac inhibited TGF-β-induced WPMY-1 cell proliferation by suppressing PCNA protein.

### 3.4 Apoptotic effect of PI extract and isolated metabolites on TGF-β-induced WPMY-1 cells

**FIGURE 5:**
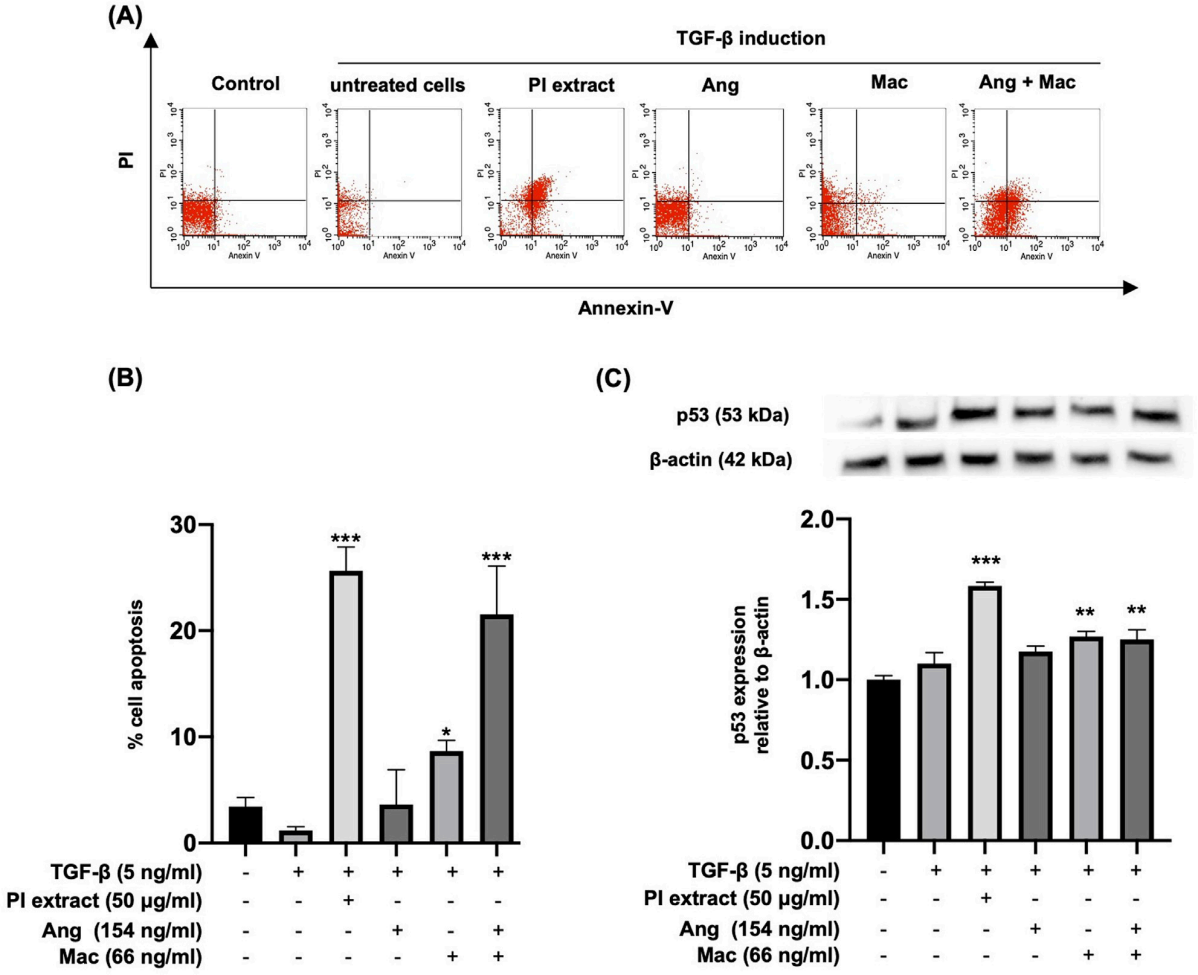
Apoptotic effects of PI extract, Ang, Mac, and Ang-Mac combination on TGF-β-induced WPMY-1 cells. WPMY-1 cells were treated with 5 ng/mL of TGF-β alone or in combination with PI extract (50 μg/mL), Ang (154 ng/mL), Mac (66 ng/mL), and Ang-Mac combination (154 + 66 ng/mL). **(A)** Flow cytometry analyses of a representative experiment. **(B)** Percentage of apoptotic cells after 24 h of treatment, expressed as mean ± SD. ***p < 0.001, and *p < 0.05 compared to TGF-β. **(C)** Western blot analysis of the p53 protein expression. The p53 protein band is normalized to β-actin, with relative density compared to the control shown as mean ± SD. ***p < 0.001, and **p < 0.01 compared to TGF-β.

The cell apoptosis assay was performed with flow cytometry, and p53 protein expression was observed by Western blot analysis (Figure 5). Flow cytometry analysis demonstrated apoptotic cells in the right upper and lower quadrants (Figure 5A). The result showed that TGF-β-induced WPMY-1 cells treated with PI extract, Mac alone, and Ang-Mac combination showed significantly increased cell apoptosis compared with TGF-β group (Figure 5B). Western blot analysis was employed to determine whether the apoptotic effect was due to the upregulation of the p53 protein. The expression of p53 was significantly increased in the treatment with PI extract (50 μg/mL), Mac (66 ng/mL), and Ang-Mac combination, whereas Ang alone and did not affect the p53 protein expression, compared to the TGF-β group (Figure 5C). The results summarized the effect of PI extract, Mac, and Ang-Mac combination on cell apoptosis, which occurs through the upregulation of the p53 protein.

### 3.5 Effects of extract, isolated metabolites on PI3K/AKT protein localization and expression in TGF-β-induced WPMY-1 cells

**FIGURE 6:**
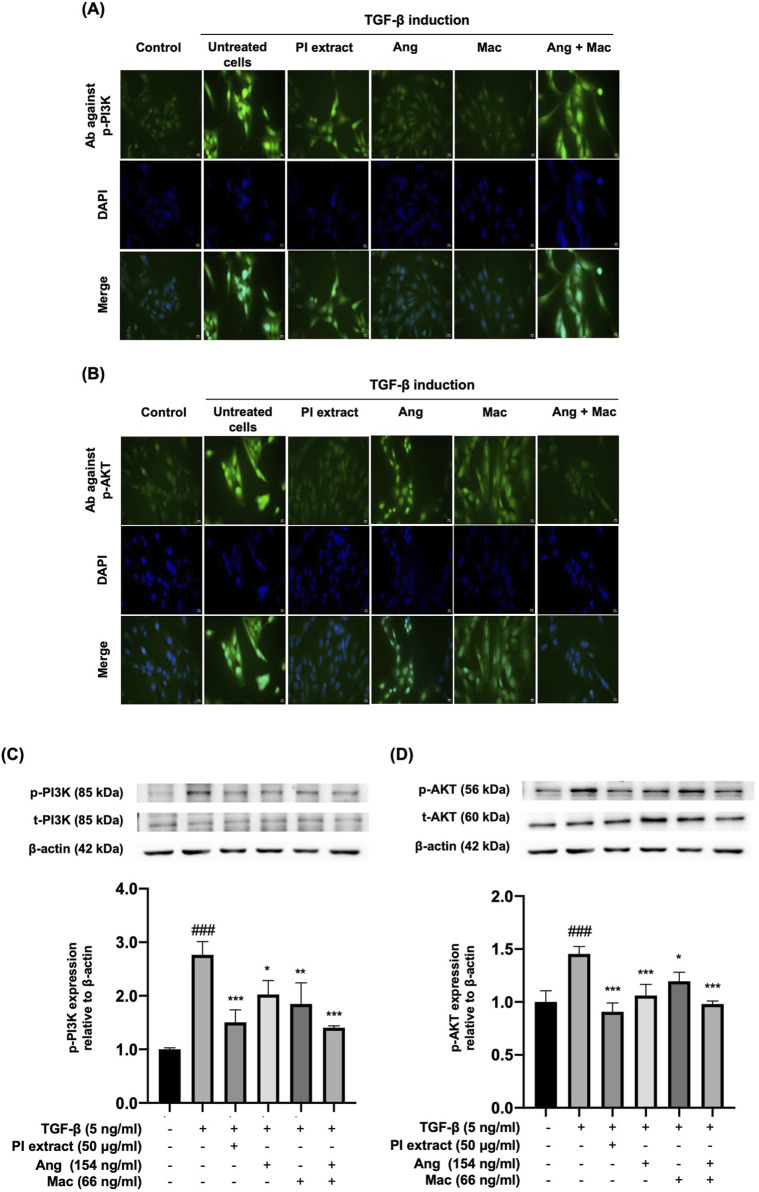
Effects of PI extract and isolated metabolites on the localization and the expression of PI3K and AKT proteins in TGF-β-induced WPYM-1 cells. The cells were incubated for 24 h in a culture medium containing TGF-β (5 ng/mL), PI extract (50 μg/mL), Ang (154 ng/mL), Mac (66 ng/mL), and Ang-Mac combination (154 + 66 ng/mL). **(A)** Immunofluorescence images reveal green signals of p-PI3K and **(B)** p-AKT protein expression in the nucleus, while blue represents nuclear staining with DAPI, scale bar, 50 μm, magnification ×400. **(C)** Protein bands of p-PI3K and **(D)** p-AKT are normalized to β-actin and shown as relative density compared to the control presented as mean ± SD, ^###^ *p* < 0.001 compared with control, ***p < 0.001, **p < 0.01, and *p < 0.05 compared with TGF-β treated group.

The effect of the PI extract, Ang, Mac, and Ang-Mac combination on the protein localization and expression in PI3K/Akt signaling pathway by indirect immunofluorescence and Western blot at the doses of 50 μg/mL, 154 ng/mL, 66 ng/mL, and 154 ng/mL + 66 ng/mL, respectively (Figure 6). The immunofluorescence figures illustrated the p-PI3K and p-AKT protein localization in WPMY-1 cells. They revealed the strong green color of the proteins expressed in the nucleus of TGF-β treated cells. Moreover, the nuclear localization of the p-PI3K proteins disappeared when the cells were treated with Ang and Mac, and the nuclear translocation was slightly inhibited in PI extract and Ang-Mac combination treated cells (Figure 6A). On the other hand, PI extract and Ang-Mac combination showed strongly inhibited p-AKT protein localization in the nucleus of the cells. At the same time, Ang and Mac individuals demonstrated slightly suppressed nuclear translocation of the protein (Figure 6B). Then, the effects of PI extract and metabolites on TGF-β-induced WPMY-1 cells through the PI3K/AKT signaling pathway were confirmed using a Western blot. Results showed the expression of p-PI3K and p-AKT proteins increased significantly in the TGF-β-induced cells compared to control. The p-PI3K protein expression was significantly inhibited in the treatment with the PI extract, Ang, Mac, and Ang-Mac combination (Figure 6C). Meanwhile, TGF-β induced p-AKT protein expression was significantly downregulated by the PI extract, Ang, Mac, and Ang-Mac combination (Figure 6D). Additionally, the findings revealed that the PI extract showed a similar inhibitory effect to that of the combination of Ang-Mac on p-PI3K and p-AKT protein expression.

## 4 Discussion

BPH arises due to loss of the imbalance between cell proliferation and apoptosis or cell death in prostate tissue (Roehrborn, 2008). α-adrenoreceptor antagonists and 5-α-reductase inhibitors are involved in the current pharmaceutical targets for BPH, which often result in various side effects, including cardiovascular issues, acute urinary retention, and sexual and ejaculatory dysfunction (Nicholson and Ricke, 2011). In recent years, interest in safe and affordable complementary and alternative medicine has grown for the long-term management of BPH. In Europe, the use of alternative and herbal medicine is reported up to 50% of prescriptions of BPH (Keehn et al., 2016). *Pterocarpus indicus* is being drawn attention for investigation of its biological activities attributed to its possessing diverse secondary metabolites like procyanidin type coumarins, tannins, flavonoids, polyphenols, and triterpenoids (Sichaem et al., 2020). In the previous studies, the extract of *P. indicus* showed antiviral activity (Dewi et al., 2018), analgesic effect (Rahman et al., 2021), antioxidant activity (Hartati et al., 2016), anti-inflammatory activity (Cha et al., 2016), and nephroprotective effect (Saputri et al., 2017). Additionally, the methanolic extract of *P. indicus* had alleviated the BPH (Yang et al., 2020). However, there has been a lack of investigation into the specific metabolites of *P. indicus* that may be responsible for this beneficial effect. Therefore, this study carried out antiproliferative-guided isolation and characterization of bioactive metabolites in the prostate stromal cell line. Herein, two secondary metabolites, angolensin, and maackiain, were isolated based on their antiproliferative activity on TGF-β-induced WPMY-1 cells (Figure 1).

Angolensin, methyl deoxybenzoin, has been isolated from the heartwood of various *Pterocarpus* species, including *P. angolensis* (King et al., 1952), *P. indicus* (Gupta and Seshadri, 1956) and *P. Erinaceus* (Seshadri, 1972). Insufficient biological investigation has been undertaken regarding angolensin, aside from its antifungal activity (Pilotti et al., 1995), antimicrobial activity against methicillin-resistant *Staphylococcus aureu*s strains (Sato et al., 2003), and α-glucosidase inhibition activity (Feunaing et al., 2023). Maackiain, a flavonoid analog, has been interested in recent years due to its broad range of pharmacological activities, such as anti-allergic (Mizuguchi et al., 2015), antioxidant (Ghribi et al., 2015), hair-growth promoting activity (Takahashi et al., 2016), neuroprotective (Tsai et al., 2020), antiviral (Bhuvaneswar et al., 2020), antibacterial (Li et al., 2022), anti-adipogenic (Mladenova et al., 2022), anti-inflammatory (Bai et al., 2022; Liu et al., 2021), and anti-cancer (Peng et al., 2022). Maackiain can inhibit cell proliferation and trigger apoptosis in human promyelocytic leukemia HL-60 cells (Aratanechemuge et al., 2004) and nasopharyngeal carcinoma cells (Jiang et al., 2023). According to the *in silico* approach investigation, maackiain is involved in the p53 signaling and apoptosis pathways (Sarkar et al., 2024). In this study, we also conducted network analysis of angolensin and maackiain combined target genes on BPH. The results revealed the top ten pathways, including the PI3K/AKT signaling pathway (Figure 3).

The PI3K and AKT signaling pathways are essential in orchestrating cell survival mechanisms. PI3K activates AKT through a secondary messenger, thereby inhibiting the activation of proapoptotic proteins. It leads to decreased apoptosis and increased prostate size, suggesting that PI3K/AKT activation may be linked with the development of BPH (Choi et al., 2022). Previous reports mentioned that the progression of BPH in rat model was inhibited through the downregulation of PI3K/AKT signaling pathway (Dong et al., 2013; Wang et al., 2022). Consequently, we focused on the effect of the PI extract, Ang, Mac, and a combination of Ang-Mac on cell proliferation and apoptosis through the PI3K/AKT pathway. Additionally, PCNA, an acidic nuclear protein that is recognized as a marker for the G1/S phase of the cell cycle (Waga et al., 1994), was observed to investigate the anti-proliferation effect of PI extract and the isolated compounds at the molecular level. As apoptosis represents a characteristic feature of programmed cell death, where the p53 protein assumes a pivotal role by inducing cell cycle arrest through the activation of pro-apoptotic genes and initiation of apoptotic pathways (Vousden, 2000; Bender et al., 2012), our study also examined the expression of p53.

To investigate whether the effects of Ang and Mac response to the activities of PI extract, the concentrations of metabolites equal to 50 μg/mL of PI extract were used to perform experiments. Our data showed that Ang and Mac might have additive inhibitory effects on PCNA protein expression and cell apoptosis, which had a greater effect than their individuals, and PI extract might be due to the containing of many metabolites that may antagonistically affect PCNA protein expression and had additive effect on apoptosis (Figures 4, 5). Treatment with Mac significantly increased apoptosis in WPMY-1 cells by upregulating the expression of the tumor suppressor gene P53, except when treated with Ang. The current finding on the apoptotic effect of Mac agreed with the results of a previous study (Aratanechemuge et al., 2004; Sarkar et al., 2024). Antiproliferative effect of Ang and an apoptotic effect of Mac make them potential candidates for treating BPH in future scientific investigations.

**FIGURE 7:**
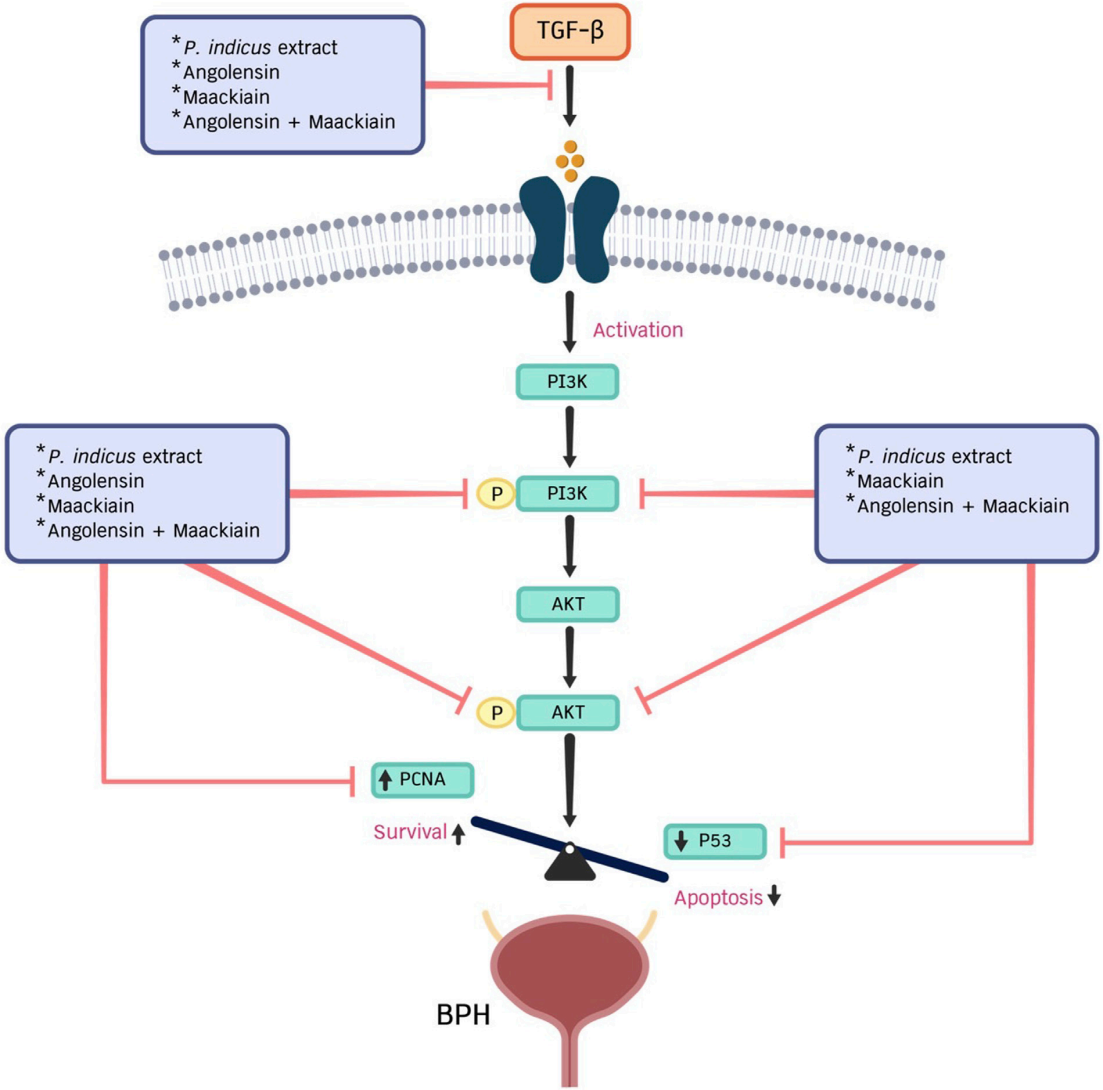
This diagram illustrates the role of PI extract, angolensin, maackiain, and a combination of angolensin and maackiain in regulating the proliferation of prostate stromal cells through PCNA, p53 proteins, and the PI3K/AKT signaling pathway.

Additionally, we observed the anti-BPH effects of the PI extract and isolated compounds on the PI3K/AKT signaling pathway. Results revealed the effects of the PI extract on the suppression of p-PI3K and p-AKT were similar to the combination of Ang-Mac, suggesting that these two metabolites are responsible for the effect of the PI extract (Figure 6). In conclusion, we can summarize that both Ang and Mac, isolated from PI extract, serve as active metabolites for inhibiting cell proliferation and inducing cell apoptosis in TGF-β-induced prostate stromal cells via the PI3K/AKT signaling pathway. Ang served the strong anti-proliferation effect of the PI extract by suppressing PCNA protein expression (Figure 7). In contrast, Mac serves the significantly induced cell apoptosis via the protein expression results regarding the PI3K/AKT signaling pathway, which agrees with the network analysis prediction. However, in this study we investigated network analysis and *in vitro* experimentation to explore the effects of PI extract, Ang, Mac, and Ang-Mac combination in TGF-β-induced WPMY-1 cells. Therefore, further exploration of other potential mechanisms involved in BPH, and different BPH cell lines are needed to validate the findings.

*Pterocarpus indicus* extract demonstrated no significant acute toxicity in both murine and brine shrimp models. In mice, no mortality occurred at doses up to 18,000 mg/kg, with an LD_50_ exceeding 5,000 mg/kg, while brine shrimp showed low toxicity (LC_50_ = 23.6 mg/mL) (Angelina et al., 2014). Similarly, maackiain showed no hepatotoxicity, mutagenicity, or cytotoxicity *in silico* (Abera et al., 2024), and *in vivo* testing confirmed no mortality in mice up to 2000 mg/kg (Guo et al., 2021). However, the safety of angolensin remains unexplored *in vivo*. To address this, we used Protox III for *in silico* toxicity prediction, which classified angolensin as safe (class 4) with an LD_50_ of 2000 mg/kg (Supplementary Table S5). Overall, while *P. indicus* extract and maackiain appear to have low acute toxicity, the data remain limited, especially regarding chronic exposure and dose escalation. Angolensin’s safety has not been confirmed in comprehensive *in vivo* models. Therefore, more extensive toxicological studies, including human trials and long-term effects on vital physiological systems, are essential to establish the safety profile of PI extract, maackiain, and angolensin before their clinical application.

## 5 Conclusion

Overall, angolensin and maackiain were isolated from the PI extract based on their antiproliferative activity on TGF-β-induced WPMY-1 cells. Additionally, these two metabolites could be used to standardize PI extract in future investigations. As supported by *in silico* and *in vitro* studies, PI extract, angolensin, and maackiain may be promising agents for treating BPH. Further research is recommended to confirm indications for BPH treatment via different cell models and other possible pathways, dosages, and safety profiles.

## Data Availability

The original contributions presented in the study are included in the article/Supplementary Material, further inquiries can be directed to the corresponding author.
