# Supplementary material for: Bioassay-guided isolation of two antiproliferative metabolites from Pterocarpus indicus Willd. against TGF-β-induced prostate stromal cells (WPMY-1) proliferation via PI3K/AKT signaling pathway
Source: Front Pharmacol. 2024 Oct 3;15:1452887. doi: 10.3389/fphar.2024.1452887 (PMC11483373; doi:10.3389/fphar.2024.1452887)
Supplement: Supplementary file 1 [file DataSheet1.pdf]

Supplementary Material

Bioassay-guided isolation of two antiproliferative metabolites from *Pterocarpus indicus* Willd. against TGF-β-induced prostate stromal cells (WPMY-1) proliferation via PI3K/AKT signaling pathway

San Yoon Nwe [1,2,3], Tamonwan Uttarawichien [1,2], Teerawat Boonsom [1,2], Wisuwat Thongphichai [1,2], Peththa Wadu Dasuni Wasana [4,5], Boonchoo Sritularak [1,6], Witchuda Payuhakrit [7], Suchada Sukrong [2,8], Pasarapa Towiwat [4,9*]

[1]Department of Pharmacognosy and Pharmaceutical Botany, Faculty of Pharmaceutical Sciences, Chulalongkorn University, Bangkok 10330, Thailand

[2]Center of Excellence in DNA Barcoding of Thai Medicinal Plants, Chulalongkorn University, Bangkok 10330, Thailand

[3]Herb Guardian Co., Ltd., Nonthaburi 11120, Thailand

[4]Animal Models of Chronic Inflammation-associated Diseases for Drug Discovery Research Unit, Chulalongkorn University, Bangkok 10330, Thailand

[5]Department of Pharmacy, Faculty of Allied Health Sciences, University of Ruhuna, Galle 80000, Sri Lanka

[6]Center of Excellence in Natural Products for Ageing and Chronic Diseases, Chulalongkorn University, Bangkok 10330, Thailand

[7]Department of Pathobiology, Faculty of Science, Mahidol University, Bangkok 10400, Thailand

[8]Chulalongkorn School of Integrated Innovation, Chulalongkorn University, Bangkok 10330, Thailand

[9]Department of Pharmacology and Physiology, Faculty of Pharmaceutical Sciences, Chulalongkorn University, Bangkok 10330, Thailand

* Corresponding author: Pasarapa Towiwat

Tel.: +662-218-8326

Address: Department of Pharmacology and Physiology, Faculty of Pharmaceutical Sciences, Chulalongkorn University, Bangkok 10330, Thailand

E-mail: pasarapa.c@chula.ac.th

1.1 Supplementary Figures

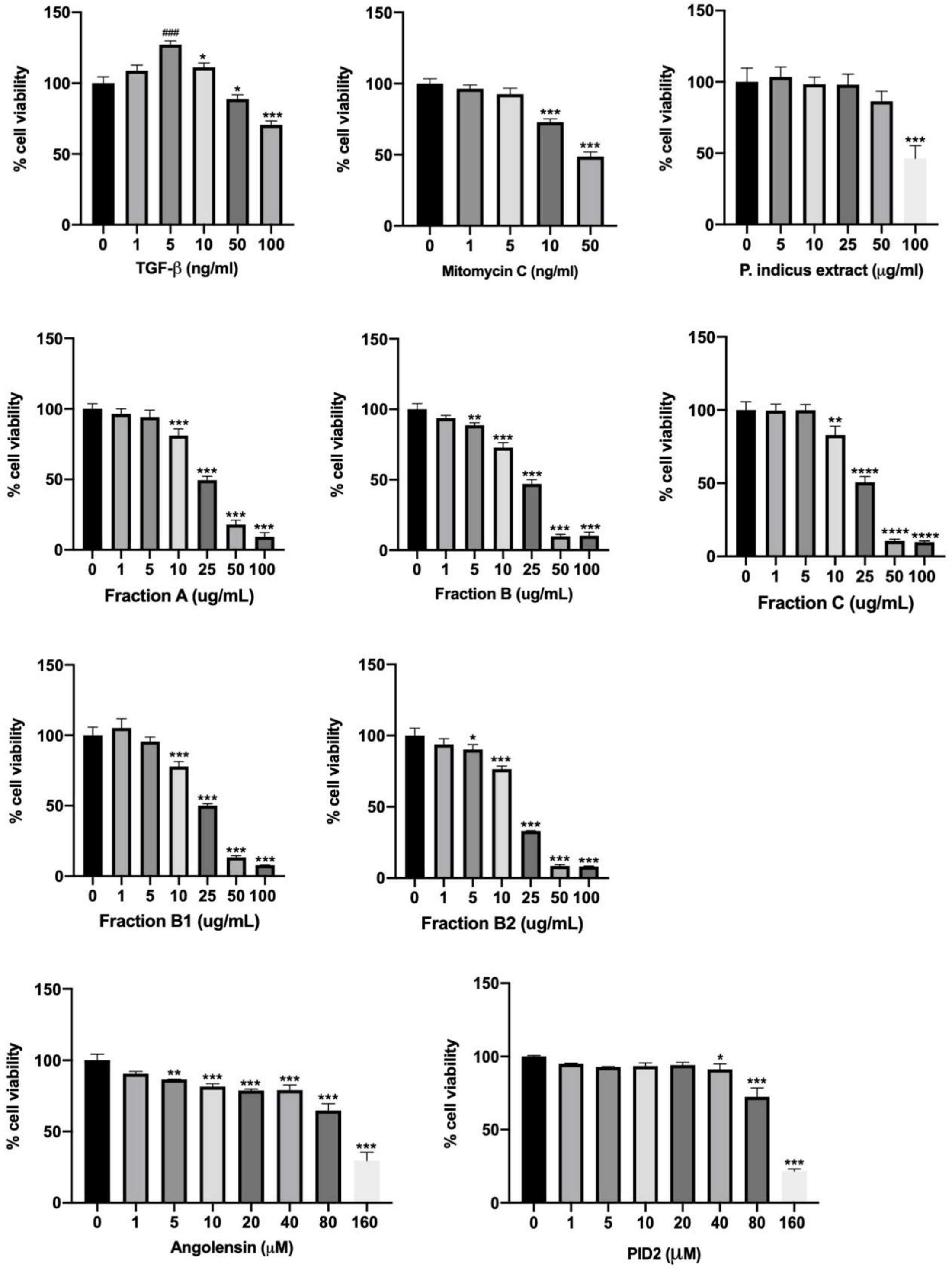

Supplementary Figure 1. Cytotoxicity activity of TGF-β, mitomycin, PI extract, fraction (A-C), fraction (B1-B2), angolensin and macckiain against WPMY-1 cells. Data are expressed as the means±SD (***P < 0.001).

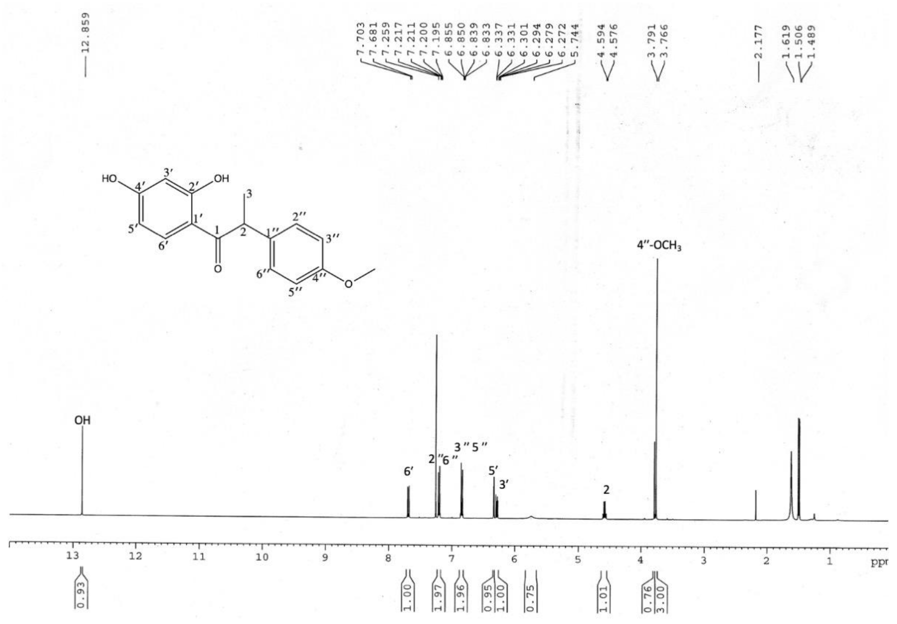

Supplementary Figure 2. ^{1}H-NMR (400 MHz, $CDCL_3$) spectrum of angolensin (ranging from 1.49-12.86).

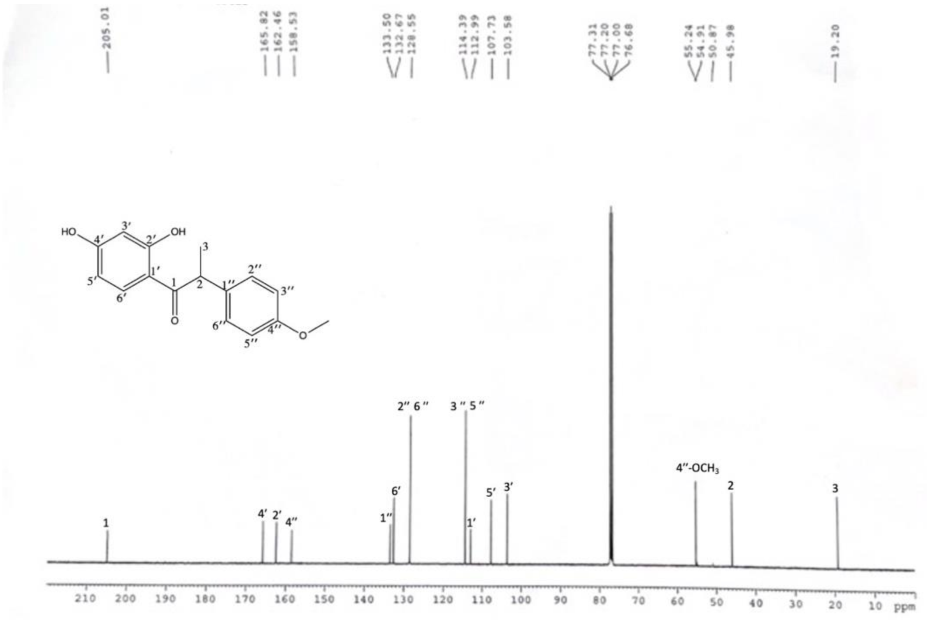

Supplementary Figure 3. ^{13}C-NMR (100 MHz, $CDCL_3$) spectrum of angolensin.

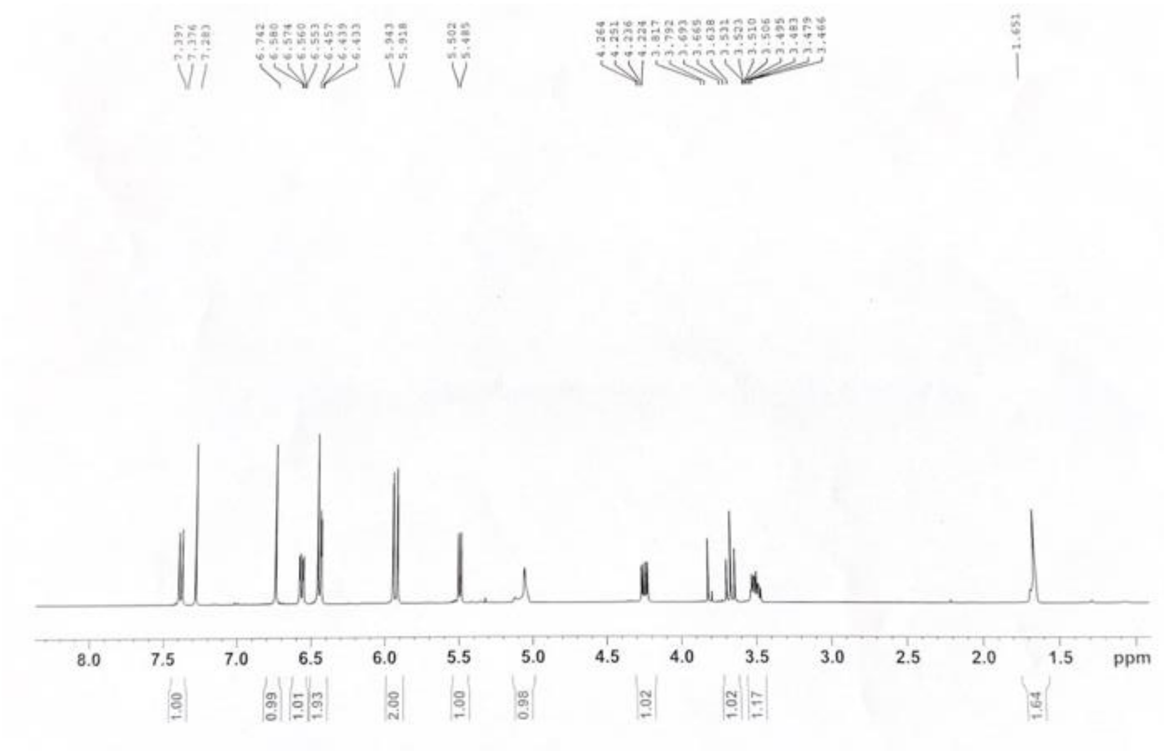

Supplementary Figure 4. ^{1}H-NMR (400 MHz, $CDCL_3$) spectrum of maackiain (ranging from 1.5 - 8.0).

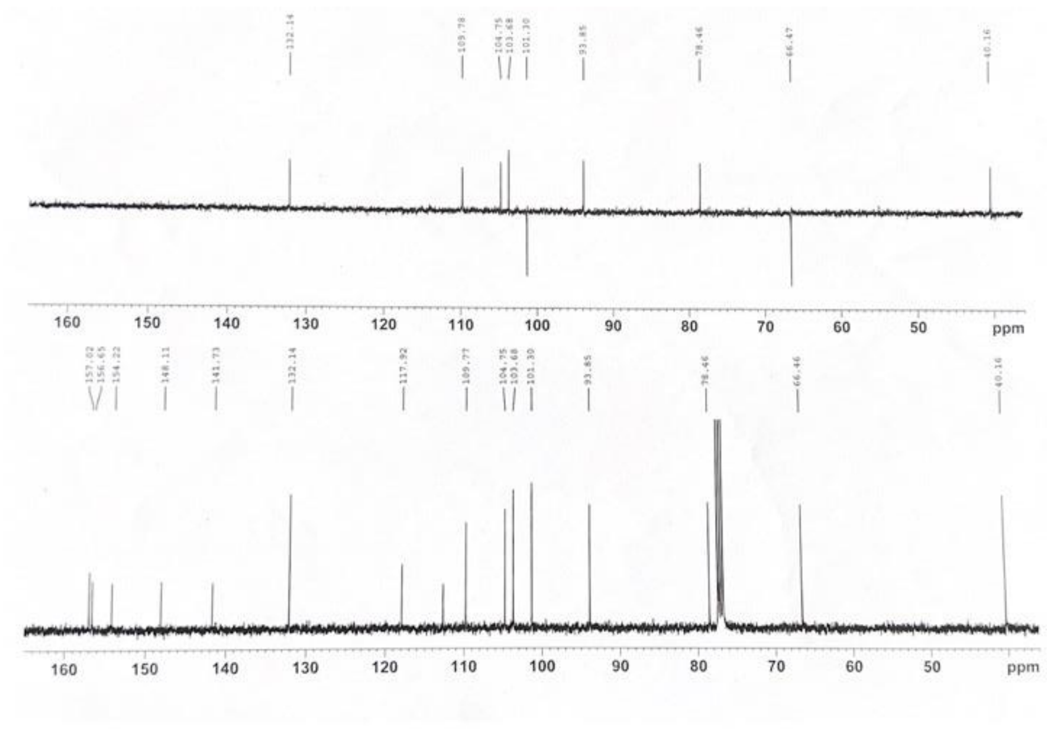

Supplementary Figure 5. ^{13}C-NMR (100 MHz, $CDCL_3$) spectrum of maackiain.

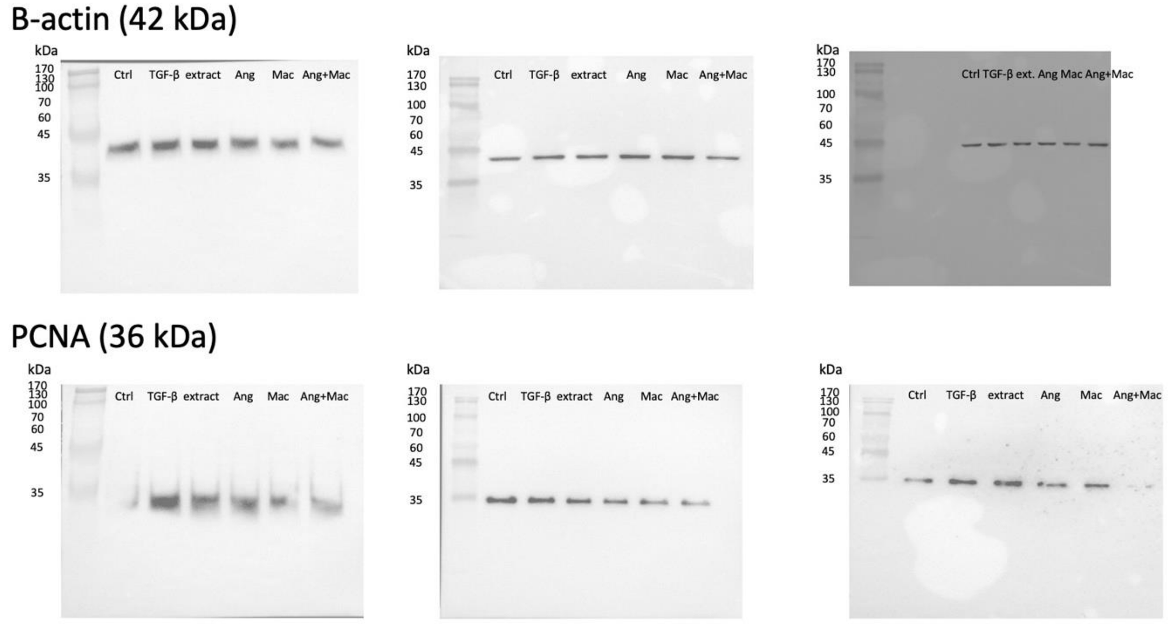

Supplementary Figure 6. Western blot analysis of B-actin and PCNA expression in three biological replicates.

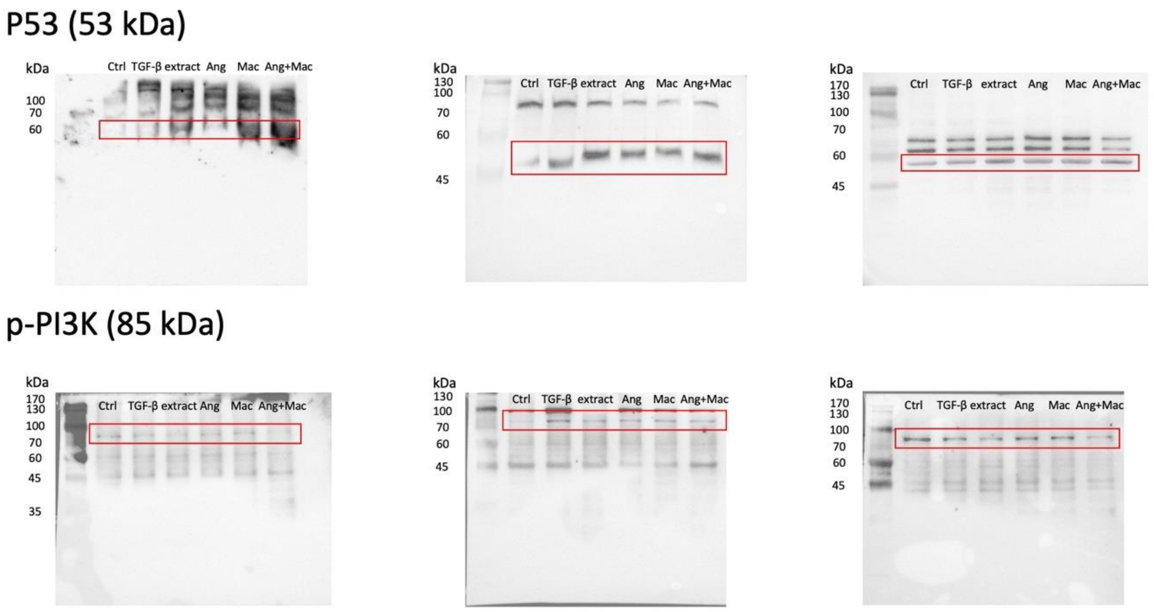

Supplementary Figure 7. Western blot analysis of p53 and p-PI3K expression in three biological replicates.

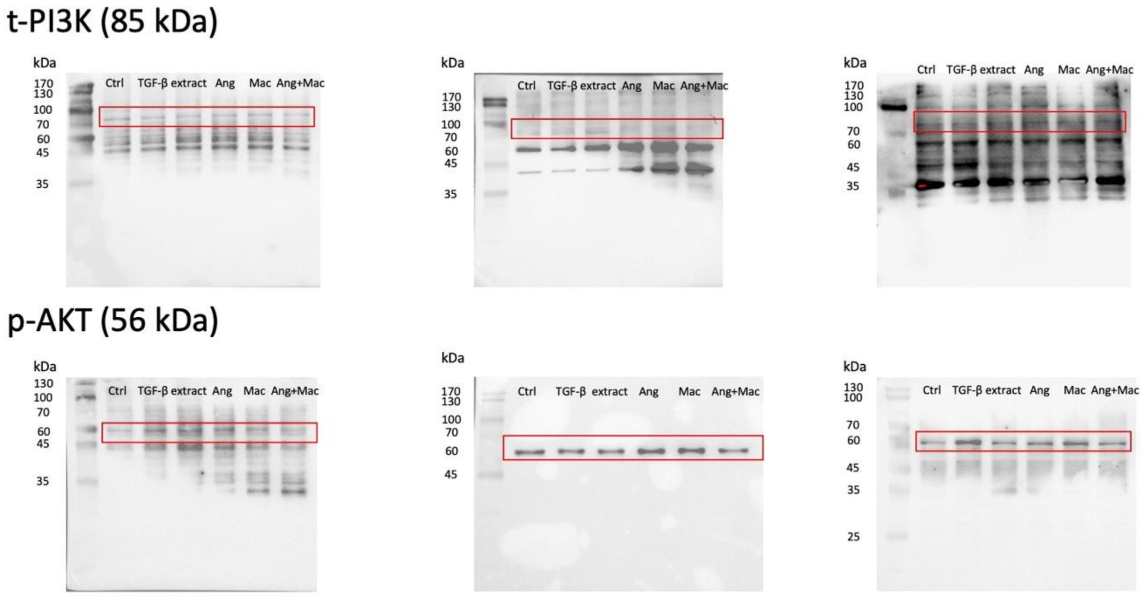

Supplementary Figure 8. Western blot analysis of t-PI3K and p-AKT expression in three biological replicates.

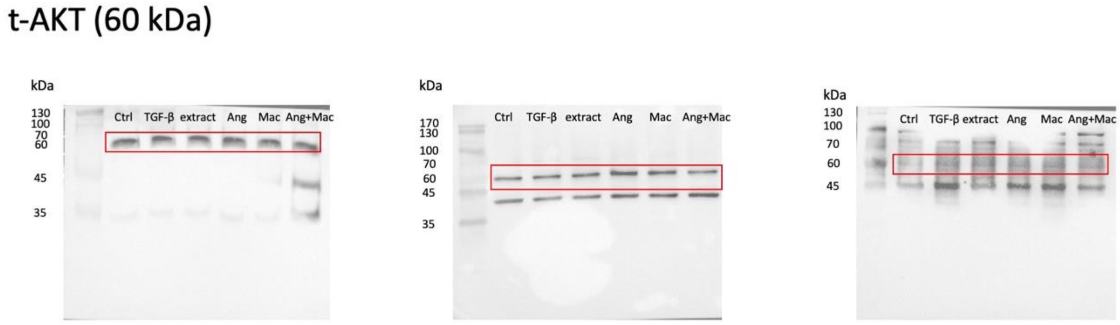

Supplementary Figure 9. Western blot analysis of t-AKT expression in three biological replicates.

1.2 Supplementary Tables

Supplementary Table 1. ^{1}H-NMR (δ_H, *J*) and ^{13}C-NMR (δ_C) spectral data obtained in this study for PI-B1 in comparison to those of a previous report.

Position	Angolensin in $CDCL_3$ (Salakka & Wähälä, 1999)		PI-B1 in $CDCL_3$	
	**δ_C**	**δ_H**	**δ_C**	**δ_H**
1	205.4		205.01	
2	45.9	4.57 (q, *J* = 6.9 Hz)	45.9	4.57 (q, *J* = 6.8 Hz)
3	19.2	1.49 (d, *J* = 6.9 Hz)	19.2	1.49 (d, *J* = 6.8 Hz)
1′	112.8		112.9	
2′	162.8		162.4	
3′	103.5	6.27 (s)	103.5	6.27 (d, *J* = 8.8 Hz)
4′	165.6		165.8	
5′	108.1	6.33 (d, *J* = 8.5 Hz)	107.7	6.33 (d, *J* = 8.8 Hz)
6′	132.8	7.68 (d, 8.5 Hz)	132.6	7.68 (d, *J* = 8.8 Hz)
1″	133.5		133.5	
2″	128.6	7.20 (d, *J* = 8.5 Hz)	128.5	7.20 (d, *J* = 8.8 Hz)
3″	114.4	6.84 (d, *J* = 8.5 Hz)	114.3	6.84 (d, *J* = 8.8 Hz)
4″	158.4		158.5	
5″	114.4	6.84 (d, *J* = 8.5 Hz)	114.3	6.84 (d, *J* = 8.8 Hz)
6″	128.6	7.20 (d, *J* = 8.5 Hz)	128.5	7.20 (d, *J* = 8.8 Hz)
-OMe	55.3	3.75 (s)	55.2	3.78 (s)
OH		12.00		12.89

The chemical shift (δ) is represented in ppm
The coupling constant (J) is represented in Hz
Angolensin in $CDCL_3$ was obtained from (Salakka & Wähälä, 1999)

Supplementary Table 2. ^{1}H-NMR (δH, *J*) and ^{13}C-NMR (δ_C) spectral data obtained in this study for PI-B2 in comparison to those of previous reports.

Position	Maackiain[a] in MeOH (Peng et al., 2016)		Maackiain[b] in DMSO-d_6 (Huh et al., 2020)		PI-B2 in $CDCL_3$	
	**δ_C**	**δ_H**	**δ_C**	**δ_H**	**δ_C**	**δ_H**
1	132.5	7.53 (d, *J* = 8.82 Hz)	131.9	7.25 (d, *J* = 8.4 Hz)	132.1	7.39 (d, *J* = 8.4 Hz)
2	110.7	6.90 (dd, *J* = 8.46 Hz)	109.6	6.48 (dd, *J* = 8.4 Hz)	109.8	6.58 (dd, *J* = 8.0 Hz)
3	160.4		158.7		157.0	
4	104.0	6.83 (d, *J* = 2.22 Hz)	102.8	6.26 (d, *J* = 2.4 Hz)	103.7	6.44 (d, *J* = 2.4 Hz)
4a	157.3		156.3		156.7	
6	66.5	3.48 (m)	65.7	3.51 (m)	66.5	3.50 (m)
6a	40.5	3.77 (t , *J* = 10.62 Hz)	40.1	3.64 t, *J* = 10.6 Hz)	40.2	3.67 (t , J=12 Hz)
6b	118.7	4.25 (dd, *J* = 11.04 Hz)	118.4	4.24 (dd, *J* = 10.9 Hz)	118.0	4.24 (dd, *J* = 11.2 Hz)
7	105.3	6.84 (s)	105.3	6.96 (s)	104.8	6.74 (1H, s)
8	141.9		141.0		141.7	
9	148.3		147.4		148.1	
10	93.7	6.64 (s)	93.2	6.51 (s)	93.9	6.56 (s)
10a	154.7		153.7		154.2	
11a	79.0	5.57 (d, *J* = 7.32 Hz)	77.9	5.49 (d, *J* = 7.0 Hz)	78.5	5.49 (d, *J* = 6.8 Hz)
11b	111.8		111.2		112.0	
(-OCH_2-O)-aH	101.5	5.91 (d, *J* =1.14 Hz)	100.9	5.93 (dd, *J* = 13.0 Hz)	101.3	5.94 (d, *J* = 10 Hz)
(-OCH_2-O)-bH		5.88 (d, *J* = 1.14 Hz)		5.93 (dd, *J* = 13.0 Hz)		5.92 (d, *J* = 10 Hz)

The chemical shift (δ) is represented in ppm
The coupling constant (J) is represented in Hz
Maackiain [a] was obtained from (Peng et al., 2016)
Maackiain [b] was obtained from (Huh et al., 2020)

Supplementary Table 3. Summary of top ten GO terms associated with the biological activity of Angolensin-Maackiain combination in benign prostatic hyperplasia.

Category	Pathway ID	Pathway Description	p.adjusted	FDR
GO_BP	GO:0051897	positive regulation of protein kinase B signaling	1.19394E-15	6.10137E-16
	GO:0043405	regulation of MAP kinase activity	2.2043E-15	1.12646E-15
	GO:0071902	positive regulation of protein serine/threonine kinase activity	2.2043E-15	1.12646E-15
	GO:0043491	protein kinase B signaling	4.63739E-15	2.36984E-15
	GO:0018209	peptidyl-serine modification	8.4926E-15	4.33996E-15
	GO:0051896	regulation of protein kinase B signaling	8.4926E-15	4.33996E-15
	GO:0043406	positive regulation of MAP kinase activity	1.67812E-14	8.57569E-15
	GO:0018105	peptidyl-serine phosphorylation	2.15313E-14	1.10031E-14
	GO:0120255	olefinic compound biosynthetic process	7.46855E-13	3.81664E-13
	GO:0046777	protein autophosphorylation	8.36242E-12	4.27344E-12

GO_CC	GO:0061695	transferase complex, transferring phosphorus-containing groups	3.79771E-07	2.69039E-07
	GO:0000307	cyclin-dependent protein kinase holoenzyme complex	4.62515E-07	3.27657E-07
	GO:1902911	protein kinase complex	8.33004E-06	5.90121E-06
	GO:0019898	extrinsic component of membrane	8.33004E-06	5.90121E-06
	GO:0005942	phosphatidylinositol 3-kinase complex	2.62817E-05	1.86186E-05
	GO:1902554	serine/threonine protein kinase complex	2.67737E-05	1.89672E-05
	GO:0045121	membrane raft	7.05816E-05	5.00017E-05
	GO:0098857	membrane microdomain	7.05816E-05	5.00017E-05
	GO:0098589	membrane region	9.10102E-05	6.44739E-05
	GO:0034774	secretory granule lumen	0.000304428	0.000215664
GO_MF	GO:0004674	protein serine/threonine kinase activity	3.33537E-12	2.327E-12
	GO:0004713	protein tyrosine kinase activity	9.14179E-08	6.37799E-08
	GO:0004714	transmembrane receptor protein tyrosine kinase activity	3.60519E-07	2.51525E-07
	GO:0019199	transmembrane receptor protein kinase activity	2.42713E-06	1.69335E-06
	GO:0019207	kinase regulator activity	5.08571E-06	3.54817E-06
	GO:0070851	growth factor receptor binding	1.06178E-05	7.40774E-06

	GO:0016303	1-phosphatidylinositol-3-kinase activity	1.42384E-05	9.93379E-06
	GO:0016538	cyclin-dependent protein serine/threonine kinase regulator activity	2.49904E-05	1.74351E-05
	GO:0035004	phosphatidylinositol 3-kinase activity	2.58678E-05	1.80473E-05
	GO:0016307	phosphatidylinositol phosphate kinase activity	7.64173E-05	5.33144E-05

Supplementary Table 4. Summary of top ten significant KEGG pathway terms modulated by Angolensin-Maackiain combination in benign prostatic hyperplasia.

Pathway ID	Pathway Description	p.adjusted	FDR
hsa05215	Prostate cancer	7.64841E-22	2.63551E-22
hsa05205	Proteoglycans in cancer	1.01449E-16	3.49577E-17
hsa01522	Endocrine resistance	2.06339E-16	7.11011E-17
hsa05212	Pancreatic cancer	2.20555E-14	7.59995E-15
hsa04151	PI3K-Akt signaling pathway	2.60339E-14	8.97087E-15
hsa04917	Prolactin signaling pathway	9.02051E-14	3.10832E-14
hsa04914	Progesterone-mediated oocyte maturation	9.02051E-14	3.10832E-14
hsa05224	Breast cancer	2.06361E-13	7.11086E-14
hsa05167	Kaposi sarcoma-associated herpesvirus infection	2.26975E-13	7.8212E-14
hsa01521	EGFR tyrosine kinase inhibitor resistance	4.05984E-13	1.39896E-13

Supplementary Table 5. Computational modeling of the potential toxicity of Angolensin performed using Pro Tox-III.

**No.**	**Variables**	**Angolensin**
1	Toxicity class (1-5)	Class 4 (Harmful if swallowed)
2	Predicted LD50 (mg/kg)	2000 mg/kg
3	Hepatotoxicity	Inactive
4	Toxicity endpoints	
	A. Carcinogenicity	Inactive
	B. Immunotoxicity	Inactive
	C. Mutagenicity	Inactive
	D. Cytotoxicity	Inactive
5	Tox21-Nuclear receptor signalling pathways	
	A. Aryl hydrocarbon Receptor (AhR)	Active
	B. Androgen Receptor (AR)	Inactive
	C. Androgen Receptor Ligand Binding Domain (AR-LBD)	Inactive
	D. Aromatase	Inactive
	E. Estrogen Receptor Alpha (ER)	Active
	F. Estrogen Receptor Ligand Binding Domain (ER-LBD)	Active
	G. Peroxisome Proliferator Activated Receptor Gamma (PPAR-Gamma)	Inactive
6	Tox21-Stress response pathways	
	A. Nuclear factor (erythroid-derived 2)-like 2/antioxidant responsive element (nrf2/ARE)	Inactive

	B. Heat shock factor response element (HSE)	Inactive
	C. Mitochondrial Membrane Potential (MMP)	Active
	D. Phosphoprotein (Tumor Suppressor) p53	Inactive
	E. ATPase family AAA domain-containing protein 5 (ATAD5)	Inactive

References

Huh, J. W.; Lee, J. H.; Jeon, E., Ryu, H. W.; Oh, S. R.; Ahn, K. S.; Jun, H. S.; Ha, U. H. Maackiain, a compound derived from *Sophora flavescens*, increases IL-1β production by amplifying nigericin-mediated inflammasome activation. FEBS Open bio **2020**, *10(8)*, 1482-1491.

Peng, T.; Zhao, F.; Chen, X.; Jiang, G.; Wang, S. Chemical study of the Chinese medicine Pi Han Yao. Biomed Rep **2016**, *4(2)*, 219-222.

Salakka, A.; Wähälä, K. Synthesis of α-methyldeoxybenzoins. Journal of the Chemical Society, Perkin Transactions **1999,** *1(18)*, 2601-2604.
